# The Psychodynamic Approach During COVID-19 Emotional Crisis

**DOI:** 10.3389/fpsyg.2021.670196

**Published:** 2021-04-09

**Authors:** Ciro Conversano

**Affiliations:** Department of Surgical, Medical and Molecular Pathology, Critical and Care Medicine, University of Pisa, Pisa, Italy

**Keywords:** psychodynamic, psychodynamic approach, clinical psychology, mental health, COVID-19, COVID-19 outbreak, psychotherapy, psychodinamic psychotherapy

The psychodynamic approach views human behavior from the standpoint of unconscious motives that influence personality functioning. In contrast with a nosological approach, the emphasis is on tracing behavior to its origins, fostering a deeper understanding of what is “behind” the overt signs and symptoms of disorder (Vaillant, 1977, 1992; Kernberg, 1988; Gabbard, 2014; Sartori et al., 2017; Mazza et al., 2019). From the early stage of development, the individual experiences life in peculiar ways that will progressively determine one's identity, including attitudes, coping strategies, cognitive processes, and relational dynamics (Cramer, 2007; Fonagy et al., 2008; Di Giuseppe et al., 2019b; Rosa et al., 2019; Giovanardi et al., 2020). Psychodynamic theories have offered a remarkable contribution to the study of unconscious processes connected with physical and mental distress. This approach has helped scholars in understanding the link between body and mind, detecting cyclical relational patterns, meaning individuals' behaviors and treating psychopathologies from an emotion-focused perspective (Bornstein, 2005; Bateman and Fonagy, 2012; Luyten and Blatt, 2015; Salvatore et al., 2015; Hilsenroth et al., 2018; McCarthy et al., 2019; Salvatore, 2019).

The impact of a dynamic approach in psychotherapy is largely documented (Midgley et al., 2009; Perry and Bond, 2012; Hilsenroth and Pitman, 2019; Lo Coco et al., 2019a; Gelo et al., 2020; Gennaro et al., 2020). Process-outcome research has highlighted the effectiveness of dynamic psychotherapy in treating various mental disorder such as depression (Meystre et al., 2017; Starrs and Perry, 2018; Perry et al., 2020), anxiety (Maffei et al., 1995; Babl et al., 2019; Solomonov et al., 2019) eating disorders (Gelo et al., 2015; Lo Coco et al., 2021), pathologic addictions (Terrone et al., 2018; Frisone et al., 2020; Lo Coco et al., 2020; Giordano et al., 2021), psychotic traits (Boldrini et al., 2019, 2020), externalizing problems (Prout et al., 2018a; Hoffman and Prout, 2020), and personality disfunction (Lingiardi and Giovanardi, 2017; Goldman et al., 2018; Aafjes-van Doorn et al., 2019; Kramer, 2019; Di Giuseppe et al., 2020b; Solomonov et al., 2020). The role of therapeutic alliance as mediator on outcomes in psychotherapy has been demonstrated in several studies highlighting the need for an in-depth investigation of patient-therapist communicative exchange (Lingiardi et al., 2010; Perry, 2014; Bhatia et al., 2017; Salvatore et al., 2017; Rocco et al., 2018; Tanzilli et al., 2018; Lo Coco et al., 2019b; Leibovich et al., 2020; Zilcha-Mano et al., 2020).

## The Psychodynamic Approach Against Covid-Related Psychological Consequences

The health emergency we are experiencing due to COVID-19 has strongly influenced not only physical health but also the mental health of the general population as well as collective behavior (Gray et al., 2020; Orrù et al., 2020b; Lenzo et al., 2021). The understanding of mental health consequences must consider individual emotional responses associated with the ongoing stressful experience of the COVID-19 pandemic (Di Giuseppe et al., 2020a; Venuleo et al., 2020). Negative emotions such as fear, anger, frustration and significant economic worries are associated with higher levels of anxiety, depression, sleep disturbances, maladaptive behaviors, and psychosomatic symptoms (Conversano et al., 2020b; Franceschini et al., 2020; Parola et al., 2020). As expected, the severity of clinical conditions is observed to be higher in vulnerable groups such as children, the elderly, psychiatric patients, and front-line workers (Aafjes-van Doorn et al., 2020b; Elbay et al., 2020; Elkholy et al., 2020; Merlo et al., 2020; Shen et al., 2020; Singh et al., 2020; Orrù et al., 2021). Research has demonstrated that emotion regulation plays a key role in stress management and adaptation helping the individual to cope with discharged feelings and thoughts related to the COVID-19 crisis, communication of the final stage of the individual's life (Iasevoli et al., 2012) and, therefore, protecting them from developing clinical levels of psychological distress (Di Giuseppe et al., 2020e; Prout et al., 2020; Walker and McCabe, 2021). The role of dynamic psychotherapy is essential in moderating people's emotional reactions, although its implementation requires the adjustment of therapeutic strategies enhancing adaptation and resilience (Aafjes-van Doorn et al., 2020a; Békés et al., 2020). From this perspective, it is imperative to improve public awareness and establish adequate procedures and prompt responses of intervention.

Due to uncertainty surrounding COVID-19, collective distress and individual suffering, the psychodynamic approach may be able to consistently identify and manage stressful life-event dynamics (Afari et al., 2014; Di Giuseppe et al., 2019a) as well as fostering emotional regulation in order to prevent possible relevant factors involved in the pathogenesis of both psychological and psychosomatic syndromes (Lenzo et al., 2020; Martino et al., 2020a,b; Conversano and Di Giuseppe, 2021; Sardella et al., 2021). This is of particular relevance at present with many experiencing grief and sorrow for the loss of a family member, a reduction of freedom, changes in daily routines and fears associated with uncertainty and the intolerance of uncertainty (Conversano et al., 2020a; Orrù et al., 2020a). In response to these stressors, the individual activates unconscious defense mechanisms, psychological strategies that help in reducing the anxiety associated with the awareness of internal conflicts and externally-charged situations (Perry, 1990; American Psychiatric Association, 2013). Since defense mechanisms are hierarchically organized and own specific psychological functions, they may cause a wide number of negative consequences as well as playing a significant role as protective factors against psychological distress and psychopathological symptoms caused by the COVID-19 outbreak (Marazziti et al., 2020). The use of high-adaptive defense mechanisms promotes increased awareness of one owns feelings related to difficult life experiences and leads to better adjustment and resilience, whereas the use of immature defenses protects the self from painful feelings and thoughts at the cost of developing maladaptive affective, cognitive and relational disfunctions (Di Giuseppe et al., 2014; Perry et al., 2019). Recent studies demonstrate that specific therapeutic intervention may increase the overall defensive maturity and improve psychological well-being and adjustment (Hoffman et al., 2016; Prout et al., 2018b, 2019; Di Giuseppe et al., 2020d).

## Conclusions

In this opinion article the relevance of the implementing psychodynamic approach in the prevention of individuals' mental health during the ongoing COVID-19 crisis has been highlighted. In accordance with Marčinko et al. (2020), the inclusion of psychodynamic interventions within the public mental health emergency system is recommended, considered as an effective strategy in reinforcing the individual's well-being both during and after the COVID-19 pandemic crisis. The application of specific therapeutic interventions derived from the psychodynamic approach can enhance emotion regulation and adaptive responses as the COVID-19 pandemic progresses.

Understanding how the ongoing pandemic is influencing human reactions to such a stressful event is essential for developing *ad hoc* effective interventions. The systematic assessment of unconscious psychological aspects of personality should be promoted for the early detection of vulnerable individuals and for improving research and clinical practice toward a personalized therapeutic approach (Lingiardi and McWilliams, 2015; Lingiardi et al., 2015; Tanzilli et al., 2016; Barber and Solomonov, 2019; Talia et al., 2019; Di Giuseppe et al., 2020c; Zilcha-Mano and Ramseyer, 2020).

## Author Contributions

The author confirms being the sole contributor to this work and approves it for publication.

## Conflict of Interest

The author declares that the research was conducted in the absence of any commercial or financial relationships that could be construed as a potential conflict of interest.
